# Comparing the Efficacy and Safety of PRP and PRF on Periorbital Skin Quality: A Randomized Split‐Face Clinical Trial

**DOI:** 10.1111/jocd.70971

**Published:** 2026-06-21

**Authors:** Elham Behrangi, Roya Zeinali, Sona Zare, Abbas Dehghani, Sara Dilmaghani, Seyyedeh Tahereh Rahimi, Masoumeh Roohaninasab, Mohammad Ali Nilforoushzadeh

**Affiliations:** ^1^ Department of Dermatology, Hazrat Fatemeh Hospital, School of Medicine Iran University of Medical Sciences Tehran Iran; ^2^ Skin and Stem Cell Research Center Tehran University of Medical Sciences Tehran Iran; ^3^ Stem Cell and Regenerative Medicine Institute Sharif University of Technology Tehran Iran; ^4^ Department of Mechanical Engineering Sharif University of Technology Tehran Iran; ^5^ Skin Repair Research Center, Jordan Dermatology and Hair Transplantation Center Tehran Iran

**Keywords:** efficacy, hyperpigmentation, periorbital rejuvenation, platelet‐rich fibrin, platelet‐rich plasma, split‐face clinical trial

## Abstract

**Background:**

Infraorbital area significantly affects perceived age and patient quality of life. Although regenerative treatments such as platelet‐rich plasma (PRP) are widely used for under‐eye, evidence comparing PRP with platelet‐rich fibrin (PRF) is limited. This study aims to compare PRP to PRF for periorbital area.

**Methods:**

In this clinical trial, each participant randomly received PRP on one side and PRF on the contralateral side in three sessions. A tear‐trough‐based approach with sandwich technique was utilized. Outcomes were assessed at baseline, 3, 4, and 7 months using biometric indices (TEWL, melanin, erythema, elasticity parameters, colorimeter), standardized grading scales (hyperpigmentation, wrinkles, and hollowness, patient satisfaction scores), and ultrasonography. Statistical analysis was conducted with significance set at *p* < 0.05.

**Results:**

Twenty‐four patients completed the study (mean age 40.71 ± 10.9 years; 95.8% female). TEWL and colorimeter decreased in both groups, without statistically significant differences between groups (*p* > 0.05). Melanin and erythema demonstrated a consistent decreasing trend in the PRF group beginning at Month 3, whereas both indices paradoxically increased at Month 3 in the PRP group before declining thereafter. Sonographic parameters, POH grade, wrinkle grade at 3 and 4 months, and Allergan grade at 4 months improved significantly in both groups (*p* < 0.05), with no significant differences between groups. Patient satisfaction increased in both groups and reached statistical significance between Months 3 and 4 (*p* = 0.037). Both treatments were tolerated, with no major adverse effects.

**Conclusion:**

Both PRP and PRF significantly improved periorbital hyperpigmentation, fine wrinkles, and mild hollowness. PRF demonstrated more stable reduction in melanin and erythema and showed earlier improvement in certain elasticity parameters. Neither treatment provided sufficient correction for severe hollowness. Repeated sessions every 3–4 months may be required to maintain results.

**Trial Registration:**

Iranian Registry of Clinical Trials (IRCT): IRCT20200127046282N51

## 1 Introduction

The periorbital or under‐eye region is a central feature of the face and plays a major role in overall facial aesthetics [1]. Because the skin in this area is thin and structurally delicate, even minor age‐related changes can noticeably alter a person's appearance. Infraorbital darkening and hollowing are commonly interpreted as signs of fatigue, emotional distress, or aging, and these perceptions are consistent across different cultural contexts [2].

Concerns such as periorbital hollowing, pigmentation, and fine wrinkling are highly prevalent and often appear early in adulthood. Although comprehensive epidemiologic data are limited, available studies report that between roughly one‐third and nearly all individuals experience some degree of under‐eye discoloration or shadowing, with women—particularly younger women—frequently seeking treatment [3]. The psychosocial effects of these changes can be substantial, as patients often feel that their appearance does not match their actual level of health or vitality. Many individuals turn to cosmetic products to conceal under‐eye darkness, and lifetime spending on such products can be considerable [2].

The under‐eye area is uniquely challenging to treat. A broad range of therapeutic modalities is used to address periorbital skin improvement, including topical formulations, chemical peels, lasers and light devices, injectable fillers, and surgical procedures [4]. However, each approach has limitations, particularly in terms of durability, safety, or ability to induce true tissue regeneration.

In recent years, there has been growing interest in autologous regenerative treatments, reflecting patient demand for biologically based interventions that stimulate endogenous repair rather than relying on synthetic substances. Among these, platelet‐rich plasma (PRP) and platelet‐rich fibrin (PRF) have gained prominence as minimally invasive options capable of promoting dermal remodeling [5]. Both preparations are derived from the patient's own blood and contain concentrated growth factors and cytokines that support fibroblast activity, extracellular matrix production, and improved skin quality. Liquid injectable PRF is the third generation of autologous platelet concentrates. Unlike PRP, it is devoid of exogenous materials and is prepared using slower centrifugation. This results in a liquid concentrate in which fibrinogen and thrombin have not yet converted into a fibrin matrix, making it injectable, similar to PRP, but with the advantage of a longer growth factor release time [6, 7].

Although PRF has gained increasing popularity in aesthetic practice, and laboratory as well as basic studies have demonstrated its greater potential compared to PRP, clinical studies directly comparing PRP and PRF for periorbital rejuvenation remain limited. Therefore, this randomized split‐face trial aims to evaluates the efficacy of PRP and PRF on infraorbital hollowing, hyperpigmentation, fine wrinkles, patient satisfaction, and overall safety.

## 2 Methods

### 2.1 Patient Selection

Patients presenting to Hazrat Fatemeh Dermatology Clinic and the Skin and Stem Cell Research Center between June 2024 and March 2025 for under‐eye rejuvenation were screened for eligibility. Individuals aged 18 to 60 years with clinically confirmed infraorbital hyperpigmentation, hollowing, or wrinkling were included. Exclusion criteria were: any under‐eye treatment within the preceding 6 months, active local infection, current malignancy, pregnancy or breastfeeding, hematologic disorders, or use of antiplatelet or anticoagulant medications (e.g., aspirin) that could alter platelet function or coagulation. Demographic information was collected using a standardized questionnaire.

All eligible participants received detailed information about the study and provided written informed consent. The study was designed as a split‐face trial consisting of three treatment sessions at four‐week intervals. For each participant, one side of the face was randomly allocated to receive PRF and the contralateral side to PRP. Baseline under‐eye status was documented using standardized photography, biometric measurements, and ultrasonography.

### 2.2 Randomization and Blinding

Side allocation (right vs. left) for PRP or PRF treatment was determined using a computer‐generated randomization list. The split‐face design ensured that each participant served as their own control. The study was double‐blinded: both the patient and the evaluating dermatologist were unaware of which side received PRP or PRF. Due to differences in preparation time and consistency between PRP and PRF, the treating dermatologist could not be blinded.

### 2.3 Preparation of PRP and PRF

A total of 20 mL of whole blood was drawn from the antecubital vein and divided into two sterile 10‐mL tubes.

#### 2.3.1 PRF Preparation

Ten milliliters of whole blood were centrifuged at 1100 RPM (Revolutions Per Minute) for 3 min without anticoagulant (Hettich Universal 320, Germany). The upper plasma layer containing the highest platelet concentration was aspirated into a sterile 3‐mL syringe. The resultant PRF remained injectable for approximately 10 min before clot formation.

#### 2.3.2 PRP Preparation

The second 10‐mL blood sample was combined with 1.5 mL of acid citrate dextrose anticoagulant (ACD‐A) and centrifuged at 1500 RPM for 10 min at room temperature (Hettich Universal 320, Germany). Platelet‐poor plasma was discarded, and the PRP fraction was collected into a 3‐mL syringe.

**FIGURE 1:**
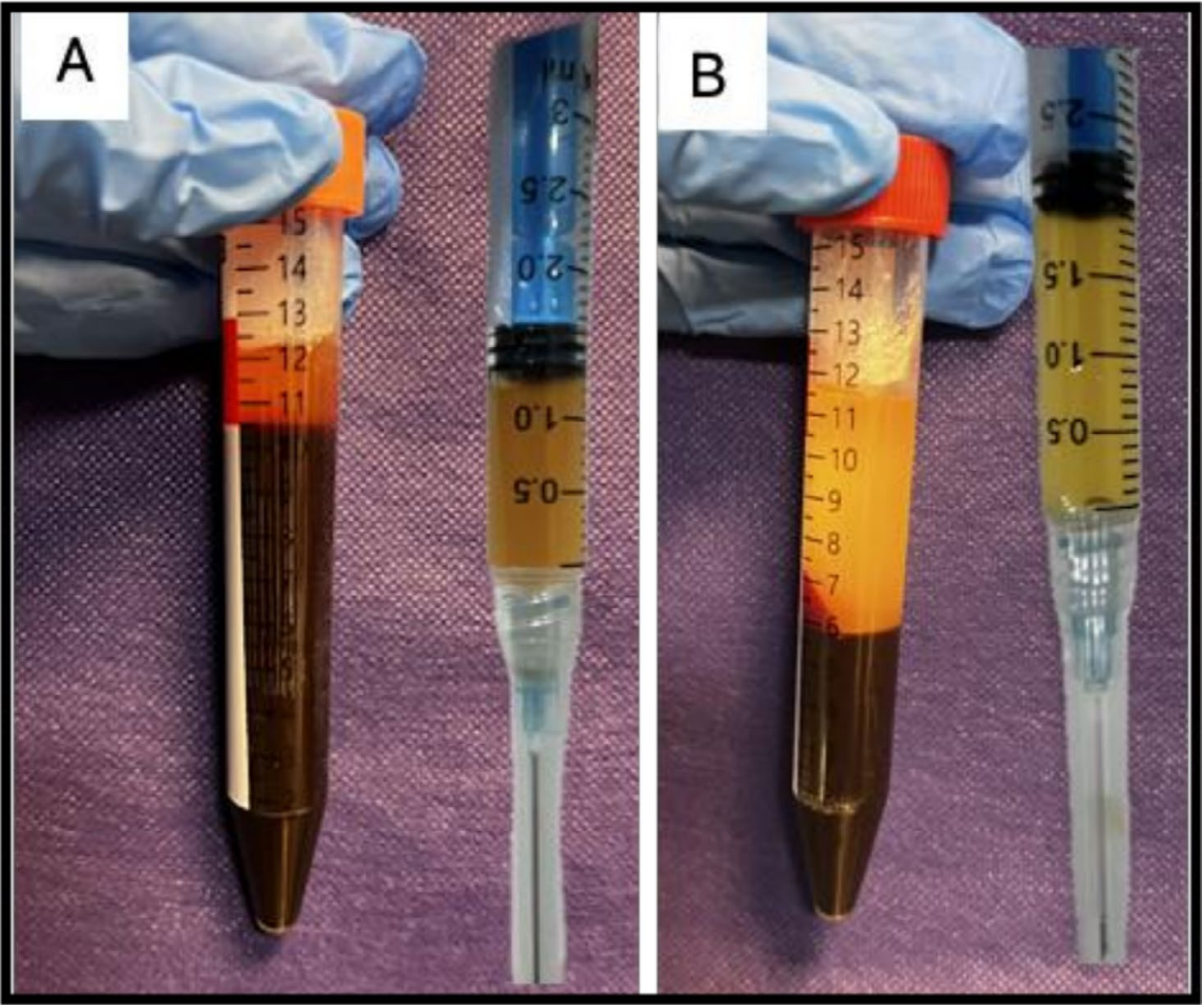
PRF (A) and PRP (B) preparations from a patient after centrifugation and extraction. Note the greater volume of PRP obtained per 10 cc of whole blood compared with PRF.

Representative PRP and PRF samples are shown in Figure 1.

### 2.4 Intervention

The treatment area was disinfected with 70% alcohol. Injections were performed using a technique similar to standard tear trough correction [8]. A vertical line was drawn from the lateral canthus, and the entry site was marked at the intersection of a perpendicular line drawn to the tear trough ligament at the level of the lateral limbus.

Approximately 0.1 mL of lidocaine was injected intradermally at the entry site for anesthesia. A 23‐gauge, 50‐mm blunt cannula was attached to the 3‐mL syringe containing PRP or PRF. The skin was gently elevated with the nondominant hand, and a pilot needle puncture was created perpendicular to the skin until the periosteum was reached. The cannula was then inserted through this entry point, with the infraorbital rim stabilized by the nondominant hand.

After advancing the cannula medially along the orbital rim, small retrograde aliquots were deposited. The process was repeated laterally. Injections were delivered in both the subdermal and deep planes, according to sandwich technique.

### 2.5 Assessment Procedures

Participants were evaluated at baseline and at 3, 4, and 7 months after treatment using the following measures:

- Biometry indices including the trans‐epidermal water loss (TEWL) by MPA‐9 Tewameter, melanin, erythema index by Mexameter, the skin elasticity (R2 (the ability to return to the original position), R5 (the elastic part of the suction phase), and R7 (the portion of the elastic recovery compared to the complete curve)) by cutometer, and color difference by colorimeter.
- **Periorbital Hyperpigmentation (POH) Grade** [9] (0–4 Scale):
  (0): Skin color not different from other facial skin areas.
  (1): Faint pigmentation of infraorbital folds.
  (2): More pronounced.
  (3): Deep dark color, all eyelids involved
  (4): Grade 3 pigmentation spreading beyond the infraorbital fold
- **Wrinkle Grading Scale** [10](Grades 0–4 with intermediate half‐grades):
  (0): no wrinkles.
  (1): a few distinct, fine wrinkles;
  (1.5): a few distinct, fine wrinkles with one or two moderate wrinkles
  (2): numerous distinct, fine wrinkles with a deep wrinkle confined to the medial side.
  (2.5): numerous distinct, fine wrinkles with a few moderate wrinkles
  (3): numerous distinct, fine wrinkles with a deep wrinkle on both medial and lateral sides and/or indistinct bags under eyes.
  (3.5): numerous distinct, fine wrinkles with three or four deep wrinkles and/or distinct bags under eyes.
  (4): numerous distinct, fine, and moderate wrinkles with numerous deep wrinkles
- **Hollowness:** Allergan Tear Trough Scale (0–4) [11].
- **Patient satisfaction:** Global Aesthetic Improvement Scale (GAIS; −1 to +3):
  (−1): worse.
  (0): no change.
  (+1): improved.
  (+2): marked improvement, but not optimal for the patient.
  (+3): Optimal cosmetic result for patient
- **Ultrasound Imaging:** High‐frequency ultrasonography was performed at baseline and Month 4 to measure epidermal, dermal, and full‐thickness skin layers and tissue density.

### 2.6 Statistical Analysis

Statistical analyses were conducted using SPSS version 26. Normality of continuous data was assessed using the Kolmogorov–Smirnov test. Normally distributed variables were summarized as mean ± SEM, and non‐normally distributed variables as median (interquartile range). Categorical variables were presented as frequencies and percentages.

For paired comparisons, paired *t*‐tests were used for normally distributed data and Wilcoxon signed‐rank tests for non‐normal data. For repeated measurements across time points, repeated‐measures ANOVA was used for normal distributions and the Friedman test for non‐normal distributions. A *p*‐value < 0.05 was considered statistically significant.

## 3 Results

A total of 25 patients were enrolled in the study. One patient was lost to follow‐up. The mean age of participants was 40.71 ± 10.9 years. Out of 24 participants, 23 patients (95.8%) were female and only one (4.2%) was male.

### 3.1 Biometric Assessment

As shown in Table 1, among the biometric indices of the PRF group, mean colorimeter changes between baseline and 7 months (*p* = 0.048) demonstrated statistically significant improvement, although an increasing trend was observed at all follow‐up time points. Although a decreasing trend was observed at every follow‐up in transepidermal water loss (Tewameter), the change was not statistically significant. A decrease in melanin was observed at Months 3 and 4; however, this reduction was not statistically significant. Erythema decreased at all follow‐up visits but did not reach statistical significance. Among the cutometry indices, R2 (gross elasticity) did not show improvement. Increasing trends in R5 (net elasticity) and R7 were observed at every follow‐up; however, this increase was not statistically significant. Sonographic indices, including complete thickness, dermal thickness, and complete, epidermal, and dermal density, all improved significantly after 4 months (*p* < 0.001, *p* < 0.001, *p* < 0.001, *p* = 0.022, and *p* < 0.001, respectively).

**TABLE 1:** The intra‐group comparison of changes in variables related to hyperpigmentation and physician and patient satisfaction in the two PRF and PRP sides before, 3, 4, and 7 months after treatment.

Characteristics	*p* ^overall^	*p* ^1–2^	*p* ^1–3^	*p* ^1–4^	*p* ^2–3^	*p* ^2–4^	*p* ^3–4^
Tewameter							
PRF Side	0.095*	0.284*	0.422*	0.101*	0.912*	0.697*	1.000*
PRP Side	0.244*	1.000*	0.956*	0.175*	0.905*	0.294*	0.994*
Melanin							
PRF Side	0.610*	1.000*	0.989*	0.908*	0.782*	0.857*	1.000*
PRP Side	0.053*	0.353*	1.000*	0.949*	0.349*	0.147*	0.410*
Erythem							
PRF Side	0.367*	0.954*	1.000*	0.907*	0.905*	0.971*	0.709*
PRP Side	0.685*	0.951*	1.000*	1.000*	0.862*	0.918*	0.999*
Colorimeter							
PRF Side	**0.008** *	0.217*	0.083*	**0.048** *	0.545*	0.286*	0.744*
PRP Side	**0.019** *	0.967*	0.200*	0.108*	0.057*	**0.022** *	0.535*
Cutometer R2							
PRF Side	0.174*	0.618*	0.680*	1.000*	1.000*	0.485*	0.728*
PRP Side	**0.001** *	**0.002** *	**0.008** *	0.833*	1.000*	0.172*	0.401*
Cutometer R5							
PRF Side	0.140*	0.730*	1.000*	0.522*	0.431*	0.983*	0.440*
PRP Side	0.635*	0.948*	0.896*	0.845*	1.000*	0.999*	0.999*
Cutometer R7							
PRF Side	0.202**	0.913**	0.435**	1.000**	0.902**	0.914**	0.725**
PRP Side	0.207**	0.957**	0.440**	1.000**	0.424**	0.994**	0.848**
Complete thickness							
PRF Side	—	—	**< 0.001** ***	—	—	—	—
PRP Side	—	—	**< 0.001** ***	—	—	—	—
Epidermal thickness							
PRF Side	—	—	0.295***	—	—	—	—
PRP Side	—	—	0.274***	—	—	—	—
Dermal thickness							
PRF Side	—	—	**< 0.001** ***	—	—	—	—
PRP Side	—	—	**0.015** ***	—	—	—	—
Complete density							
PRF Side	—	—	**< 0.001** ***	—	—	—	—
PRP Side	—	—	**< 0.001** ***	—	—	—	—
Epidermal density							
PRF Side	—	—	**0.022** ***	—	—	—	—
PRP Side	—	—	**< 0.001** ***	—	—	—	—
Dermal density							
PRF Side	—	—	**< 0.001** ***	—	—	—	—
PRP Side	—	—	**< 0.001** ***	—	—	—	—
POH Grade							
PRF Side	**< 0.001** **	**0.002** **	**< 0.001** **	**< 0.001** **	**< 0.001** **	**0.013** **	0.710**
PRP Side	**< 0.001** **	**0.013** **	**< 0.001** **	**< 0.001** **	**< 0.001** **	**0.006** **	0.983**
Wrinkle Grade							
PRF Side	**< 0.001** **	**0.002** **	**< 0.001** **	**< 0.001** **	**0.001** **	**0.002** **	0.943**
PRP Side	**< 0.001** **	**0.005** **	**< 0.001** **	**< 0.001** **	**< 0.001** **	**< 0.001** **	0.710**
Allergan Grade							
PRF Side	**< 0.001** *	0.227*	**< 0.001** *	**< 0.001** *	**0.001** *	**0.021** *	0.655*
PRP Side	**< 0.001** *	0.655*	**< 0.001** *	**0.001** *	**< 0.001** *	**0.021** *	0.227*
Patient GIAS							
PRF Side	**0.010** *	—	—	—	**0.037** *	0.089*	1.000*
PRP Side	**0.027** *	—	—	—	**0.037** *	0.280*	0.760*

*Note:* *p*‐values < 0.05 are presented in bold.

Abbreviations: 1, before treatment; 2, 3 months later; 3, 4 months later; 4, 7 months later.

* *p*‐value by repeated measures ANOVA test.

** *p*‐value by the Friedman test.

*** *p*‐value by paired samples *t*‐test.

Among the biometric indices of the PRP group, a decreasing trend was observed at every follow‐up in transepidermal water loss (Tewameter), although this decrease was not statistically significant. Interestingly, an increase in the melanin index was observed at Month 3; although a decreasing trend was observed thereafter, the change was not statistically significant. Similarly, the erythema index increased at Month 3, followed by a decreasing trend thereafter. However, colorimetry increased at all follow‐ups and was statistically significant only between Months 3 and 4 (*p* = 0.022). Interestingly, Cutometer R2 decreased significantly between baseline and 3 and 4 months post‐treatment (*p* = 0.002 and *p* = 0.008). R5 and R7 showed an increasing trend, but these changes were not statistically significant. Complete thickness, dermal thickness, complete density, epidermal density, and dermal density all showed significant improvements after 4 months on sonographic evaluation (*p* < 0.001, *p* = 0.015, *p* < 0.001, *p* < 0.001, and *p* < 0.001, respectively).

**FIGURE 2:**
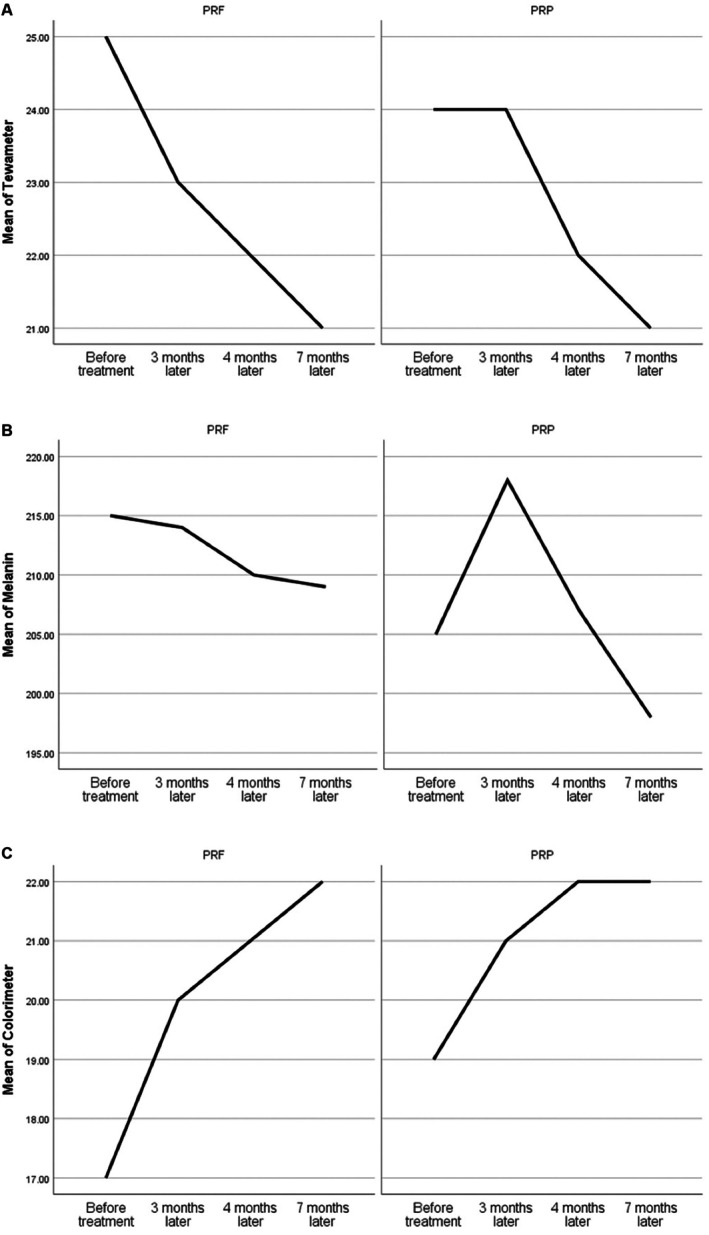
Trends in mean changes in Tewameter (A), Melanin Index (B), and Colorimeter (C) measurements in both the PRF (left) and PRP (right) groups.

Between‐group comparisons of changes are presented in Table 2. Tewameter values decreased in both groups; although this decreasing trend was greater in the PRF group, the between‐group difference was not statistically significant. Melanin levels at baseline (*p* = 0.004) were significantly higher on the PRF side compared with the PRP side. Paradoxically, melanin increased at Month 3 in the PRP group, whereas a decreasing trend began at Month 3 in the PRF group; however, the between‐group difference was not statistically significant. Similarly, the erythema index increased at Month 3 in the PRP group, followed by a decreasing trend at subsequent follow‐ups, whereas PRF demonstrated a decreasing trend in erythema throughout the follow‐up period; however, the between‐group differences were not statistically significant. Cutometer R2 and Cutometer R5 did not show significant between‐group differences. However, Cutometer R7 at 3 months (*p* = 0.022) was significantly higher on the PRF side compared with the PRP side. Among the sonographic indices, only complete density at baseline was significantly higher on the PRF side compared with the PRP side (*p* = 0.044). However, no significant between‐group differences were observed for the remaining variables (*p* > 0.05). The trends of mean changes in selected biometric indices are illustrated in Figure 2.

**TABLE 2:** The between‐group of changes in variables related to hyperpigmentation and physician and patient satisfaction in the two PRF and PRP sides before, 3, 4, and 7 months after treatment.

Characteristics	PRF side	PRP side	*p*
Tewameter			
Before treatment	24.45 (±5.5)	23.61 (±4.8)	0.196*
3 months later	22.35 (±4.9)	22.57 (±4.4)	0.075*
4 months later	21.11 (±7.4)	21.68 (±8.2)	0.391*
7 months later	21.57 (±7.4)	21.35 (±6.7)	0.750*
Melanin			
Before treatment	207.64 (±62.5)	198.49 (±60.0)	**0.004** *
3 months later	204.57 (±59.3)	209.75 (±66.9)	0.419*
4 months later	201.93 (±54.6)	203.07 (±55.6)	0.796*
7 months later	209.48 (±53.0)	198.77 (±37.0)	0.050*
Erythem			
Before treatment	363.46 (±50.8)	357.71 (±60.7)	0.426*
3 months later	359.31 (±52.3)	367.42 (±67.3)	0.364*
4 months later	366.37 (±54.7)	354.65 (±53.9)	0.119*
7 months later	352.07 (±76.0)	365.43 (±42.2)	0.407*
Colorimeter			
Before treatment	18.79 (±12.1)	20.96 (±11.9)	0.086*
3 months later	21.50 (±11.8)	22.17 (±10.7)	0.264*
4 months later	22.46 (±10.5)	23.08 (±10.2)	0.257*
7 months later	22.00 (±8.8)	22.80 (±9.0)	0.138*
Cutometer R2			
Before treatment	0.64 (±0.1)	0.65 (±0.1)	0.698*
3 months later	0.59 (±0.1)	0.57 (±0.1)	0.356*
4 months later	0.57 (±0.1)	0.57 (±0.1)	0.906*
7 months later	0.64 (±0.1)	0.63 (±0.1)	0.797*
Cutometer R5			
Before treatment	0.55 (±0.1)	0.50 (±0.1)	0.072*
3 months later	0.59 (±0.1)	0.56 (±0.1)	0.327*
4 months later	0.53 (±0.1)	0.54 (±0.1)	0.663*
7 months later	0.63 (±0.2)	0.58 (±0.2)	0.150*
Cutometer R7			
Before treatment	0.30 (0.26–0.38)	0.28 (0.27–0.34)	0.218**
3 months later	0.31 (0.29–0.37)	0.30 (0.26–0.33)	**0.022** **
4 months later	0.29 (0.26–0.32)	0.26 (0.25–0.30)	0.179**
7 months later	0.32 (0.26–0.44)	0.34 (0.24–0.37)	0.156**
Complete thickness			
Before treatment	1266.08 (±133.1)	1256.33 (±120.7)	0.741*
4 months later	1353.38 (±127.9)	1339.29 (±114.0)	0.660*
Epidermal thickness			
Before treatment	81.88 (±18.5)	74.50 (±18.0)	0.178*
4 months later	85.96 (±18.6)	93.79 (±83.1)	0.665*
Dermal thickness			
Before treatment	1184.21 (±128.9)	1181.46 (±119.9)	0.927*
4 months later	1264.33 (±124.4)	1245.13 (±142.5)	0.571*
Complete density			
Before treatment	41.35 (±13.4)	36.81 (±12.4)	**0.044** *
4 months later	49.74 (±13.9)	46.39 (±12.3)	0.298*
Epidermal density			
Before treatment	84.29 (±19.0)	78.01 (±21.5)	0.199*
4 months later	95.25 (±17.9)	91.61 (±16.9)	0.476*
Dermal density			
Before treatment	1184.21 (±128.9)	1181.46 (±119.9)	0.098*
4 months later	1264.33 (±124.4)	1245.13 (±142.5)	0.467*
POH Grade			
Before treatment	2.00 (1.00–2.00)	2.00 (1.00–2.00)	0.317**
3 months later	1.00 (1.00–2.00)	1.00 (1.00–2.00)	0.157**
4 months later	1.00 (0.00–1.00)	1.00 (0.00–1.00)	0.157**
7 months later	1.00 (0.00–1.00)	1.00 (0.00–1.00)	0.564**
Wrinkle Grade			
Before treatment	1.50 (1.00–2.50)	1.00 (1.50–2.00)	0.157**
3 months later	1.00 (1.00–1.86)	1.25 (1.00–2.00)	0.157**
4 months later	1.00 (0.00–1.00)	1.00 (0.00–1.50)	0.083**
7 months later	1.00 (0.00–1.50)	1.00 (0.00–1.50)	0.257**
Allergan Grade			
Before treatment	1.75 (±0.6)	1.75 (±0.6)	1.000*
3 months later	1.54 (±0.7)	1.67 (±0.6)	0.083*
4 months later	1.04 (±0.7)	1.08 (±0.7)	0.575*
7 months later	1.15 (±0.8)	1.25 (±0.9)	0.330*
Patient GIAS			
3 months later	1.83 (±1.0)	1.79 (±0.9)	0.328*
4 months later	2.33 (±0.6)	2.29 (±0.6)	0.575*
7 months later	2.38 (±0.7)	2.19 (±0.7)	0.104*

*Note:* *p*‐values < 0.05 are presented in bold.

* *p*‐value by Paired Samples *t*‐test.

** *p*‐value by the Wilcoxon Signed Ranks test.

### 3.2 Clinical Evaluation

Across subjective assessments of PRF group (Table 1), mean POH Grade differences improved remarkably between baseline and 3, 4, and 7 months, as well as between Months 3 and 4 and Months 3 and 7 (*p* < 0.001, *p* = 0.002, *p* < 0.001, *p* < 0.001, and *p* = 0.013, respectively). Similar significant changes were observed for Wrinkle Grade across all same time points (*p* < 0.05). Allergan Grade increase was initiated at Month 4 and significant differences were observed between Months 3 and 4 and between Months 3 and 7 (*p* < 0.001 and *p* = 0.011).

In PRP group, POH Grade improved significantly between baseline and 3, 4, and 7 months, and between Months 3 and 4 and Months 3 and 7 (*p* < 0.001, *p* = 0.013, *p* < 0.001, *p* < 0.001, and *p* = 0.006). Wrinkle Grade showed the same pattern of improvement (*p* < 0.001, *p* = 0.005, *p* < 0.001, *p* < 0.001, and *p* < 0.001). Similar to PRF, Allergan Grade showed significant differences between Months 3 and 4 and Months 3 and 7 (*p* < 0.001 and *p* = 0.011).

**FIGURE 3:**
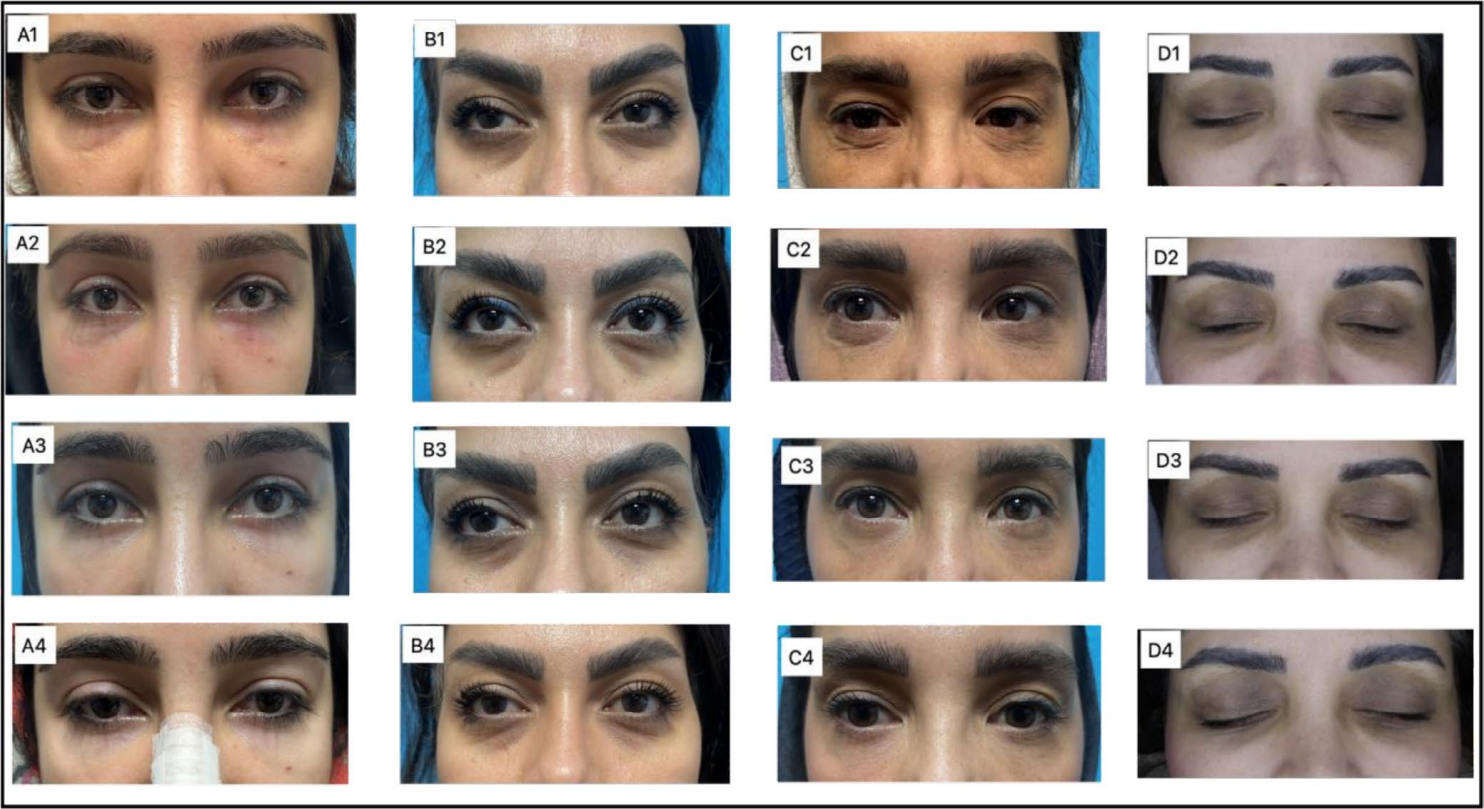
Four patients with different concerns before treatment (1), after 3 months (2), after 4 months (3), and after 7 months (4). Patient A presented with vascular hyperpigmentation and received PRF on the right side and PRP on the left. Patient B had dark circles and hollowness and received PRF on the left side and PRP on the right. Patient C presented with fine lines and dehydration and received PRF on the right side and PRP on the left. Patient D had severe pigmented dark circles and received PRF on the right side and PRP on the left.

No significant between‐group differences were observed regarding POH Grade, Wrinkle Grade, Allergan Grade at baseline or at 3, 4, or 7 months post‐treatment (*p* > 0.05) (Table 2). Figure 3 illustrates photography of some participants.

Patient GIAS showed a significant improvement between Months 3 and 4 (*p* = 0.037) and no statistically significant difference was observed between groups.

### 3.3 Safety and Tolerability

All patients described both methods as tolerable. About 47% of patients reported more pain on the PRP side, which they described as post‐injection pain. About 12.5% reported more pain on the PRF side, while the others reported no difference. No major side effects were observed. About 62.5% of patients reported swelling, which resolved within several days, with no difference between sides. Only one patient reported continuous pain on the PRP side, which resolved within 2 days.

## 4 Discussion

There has been a growing interest in regenerative approaches within aesthetic dermatology, and platelet‐based therapies such as PRP and the more recent PRF have become widely used options. While the efficacy of PRP for periorbital hyperpigmentation and age‐related changes is well documented, head‐to‐head comparisons with PRF remain limited in clinical research. This study is among the earliest randomized split‐face trials to evaluate both modalities comprehensively for under‐eye concerns.

Based on objective measurements, clinical grading, and patient perspectives, three monthly sessions of either PRP or PRF resulted in significant improvement in periorbital hyperpigmentation and fine wrinkles. Although the improvement trend was higher in the PRF group, no meaningful difference in efficacy, tolerability, or safety was detected between the two treatments. Moreover, patients more frequently described post‐injection discomfort on the PRP‐treated side.

The ability of PRP to improve dark circles is supported by extensive literature. PRP contains high concentrations of regenerative growth factors, including TGF‐β1, PDGF, EGF, IGF, and VEGF. TGF‐β1 downregulates MITF, decreases tyrosinase activity, and ultimately reduces melanin synthesis [12]. In addition to this pigment‐related effect, PRP has anti‐inflammatory properties and enhances angiogenesis, which can reduce vascular congestion contributing to periorbital darkness. Furthermore, PRP stimulates fibroblasts to synthesize collagen types I and III, elastin, and extracellular matrix proteins, leading to dermal thickening and improved optical reflectance of the skin [13]. These mechanisms are consistent with the clinical improvements observed in our study, including better hydration, reduced pigmentation severity, improved wrinkle grades, and enhanced sonographic skin parameters in the PRP‐treated areas. Current evidence supports that PRP reduces the pigmentation severity for POH [5], with good improvement and moderately effective [4].

PRF also demonstrated meaningful and sustained benefits in this study, with improvements in fine lines, pigmentation, and infraorbital hollowing that persisted through the seven‐month follow‐up. As a second‐generation platelet concentrate prepared without anticoagulants and centrifuged at a lower speed, PRF contains many of the same growth factors as PRP but releases them more gradually due to its fibrin matrix. This gradual release has been associated with more prolonged cytokine activity and potentially greater collagen synthesis [6]. However, in the current study, the clinical longevity of PRF was comparable to that of PRP, and the hypothesis that PRF provides longer‐lasting effects than PRP was not confirmed. Further studies are needed to clarify potential differences in durability between these treatments. The trend of changes observed in the current study suggests that although improvements were evident at Month 7, partial reversal of some parameters was also noted at this time point. Therefore, it may be suggested that PRP and PRF injections should be repeated every 3–4 months to achieve sustained regenerative benefits.

Clinical evidence supports PRF's effectiveness in skin rejuvenation. Hassan et al. reported significant improvement in objective and subjective skin quality parameters after three monthly intradermal facial PRF injections [14]. Majewska et al. demonstrated a measurable 5‐fold increase in lower eyelid skin density after three intradermal PRF sessions, confirmed by ultrasound imaging, along with high patient satisfaction (mean VAS 8.5) [15]. Another study by Mahmoodabadi et al. reported a significant reduction in wrinkle depth (*p* < 0.05) and an increase in tissue volume following PRF treatment using canula, as measured by 3D skin analysis [16]. Participants also experienced improved periocular hyperpigmentation and overall skin freshness. These findings align with our results demonstrating improved skin texture and under‐eye quality following PRF therapy.

Although PRF is often described as more effective for dermal quality than for pigmentation [5], our study found improvements in both hyperpigmentation and hollowness. The subjective POH scores improved substantially on both sides, and biometric assessments showed increased colorimeter values (indicating brighter skin), reduced erythema, and decreasing trends in melanin index for both groups. Increases in hydration, dermal thickness, and tissue density contributed to overall periorbital rejuvenation and reduction of shadowing and rejuvenation (Figure 3).

In contrast to our findings, Yousef et al. reported significantly superior clinical outcomes with PRF over PRP for POH after three sessions with intradermal injection, with higher response rates (*p* = 0.0001) and markedly greater patient satisfaction for PRF [17]. Several factors may explain the discrepancy. Differences in baseline severity, injection technique, depth of delivery, frequency of sessions, and reliance on subjective clinical assessments may have contributed to the stronger superiority of PRF in their study. Current literature has not established clear superiority of either treatment overall, and our findings support the emerging view that both modalities are effective for periorbital treatment [5]. Its worth mentioning that completing the seven‐month study by most patients illustrates the efficacy, and most participants demanded for continuing this procedure.

It should be noted that in the current study, although both PRP and PRF clinically reduced dark circles, as evident over the seven‐month follow‐up, biometric findings suggest that PRF provided a more stable reduction in melanin and erythema, whereas PRP was associated with a transient increase in the melanin index during the initial sessions. While there is strong evidence that PRP decreases pigmentation, the findings of the current study suggest that PRP may lead to a transient increase in the melanin index, although this increase is clinically unnoticeable. To our knowledge, there is only one case report stating that PRP may worsen pigmentation in the treatment of post‐inflammatory hyperpigmentation (PIH) and photoaging [18]. We hypothesize that PRP injections may induce a greater inflammatory response, leading to increased erythema and post‐inflammatory hyperpigmentation, while the antipigmentary effects may take longer to become apparent. Further studies are needed to determine whether this finding is incidental or represents a true effect of PRP treatment.

Overall, these findings suggest that when the primary treatment goal is reduction of pigmentation in dark circles, PRF may be more favorable than PRP. However, colorimetric measurements showed significant increases during follow‐up, indicating progressive lightening of the under‐eye area in both groups. This trend was more pronounced in the PRF group, although the between‐group difference was not statistically significant.

Cutometric evaluation demonstrated that both PRP and PRF improved skin elasticity; however, PRF resulted in earlier improvement in certain parameters. The increase in R5 indicates enhancement of the skin's pure elastic recoil. Although R2 showed a decrease, this parameter reflects both elastic and viscous components and may represent changes in viscoelastic properties rather than a true decline in elasticity. R7 showed a non‐significant upward trend, suggesting a potential improvement in biological elasticity that should be further investigated in larger studies.

**FIGURE 4:**
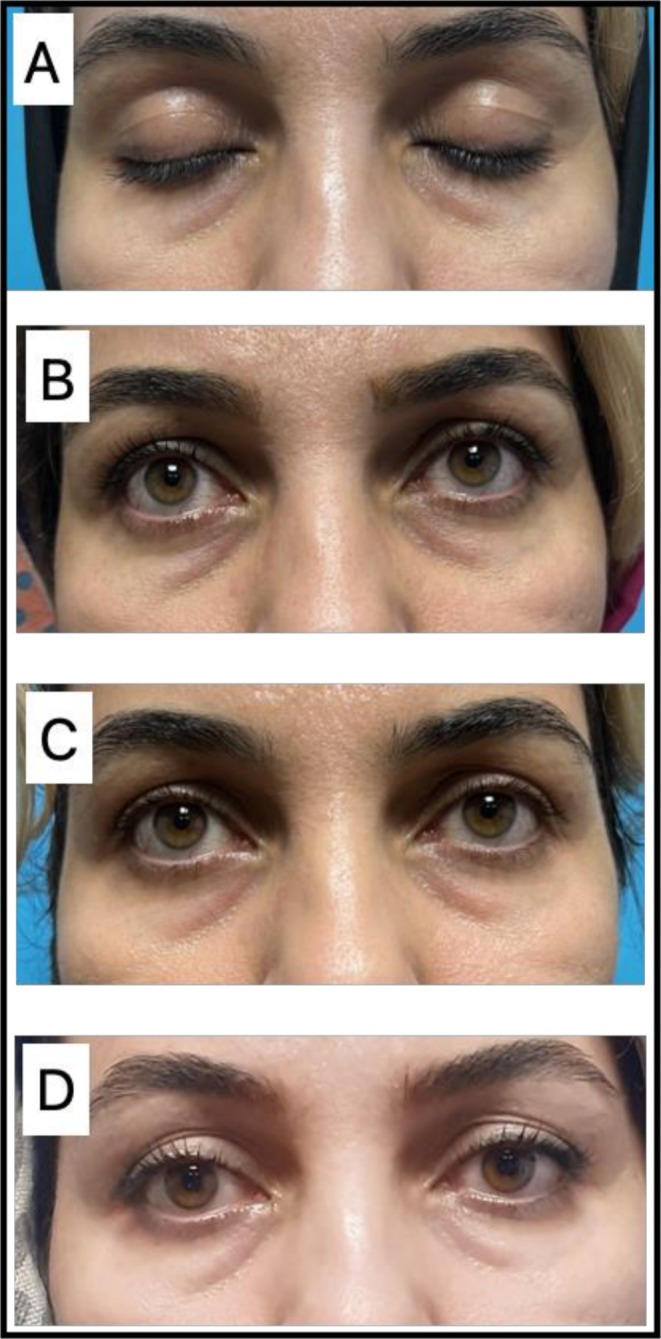
Images of a patient with severe hollowness. Although the treatment led to improved skin texture and pigmentation, no significant change was observed in the hollowness.

Although both treatments increased dermal thickness and density, their volumizing effects remained mild and insufficient for patients with moderate to severe tear‐trough deformity. According to the Allergan scale, if improvement of hollowness is the treatment goal, multiple PRP or PRF sessions are needed. This underscores that PRP and PRF primarily enhance tissue quality rather than replace lost volume (Figure 4). A case series reported that the combination of PRF with hyaluronic acid led to significant and rapid improvement in lower eyelid rejuvenation [19]. It is recommended that future trials focus on combining PRF or PRP with non‐crosslinked hyaluronic acid, not only to improve the tear trough but also to enhance the regenerative benefits of the treatment.

Cannula injection was chosen to isolate the biological effects of the platelet concentrates themselves and minimize the microtrauma associated with needle mesotherapy. Despite this, more patients reported discomfort on the PRP side, a finding consistent with reports in other medical fields (such as dental surgery) where PRF is associated with less postoperative pain and swelling [20].

Safety outcomes were favorable in both groups, consistent with the autologous nature of PRP and PRF [5]. PRF has the added advantage of avoiding anticoagulants, simplifying preparation and eliminating risks associated with additives.

This trial has several strengths. It is among the first randomized split‐face studies directly comparing PRP and PRF for under‐eye rejuvenation, controlled for individual variability, and included both subjective and objective evaluation methods, including biometry, clinical scoring, ultrasound, and patient satisfaction. All injections were performed by a single experienced provider, ensuring consistency, and the seven‐month follow‐up allowed assessment of both short‐term and intermediate‐term outcomes.

Nevertheless, limitations exist. The sample size was modest, reducing the power to detect subtle differences between PRP and PRF. Variability in PRP and PRF preparation methods across studies may influence outcomes and limit generalizability. Standardization of centrifugation protocols and multi‐center trials with larger cohorts are needed to further clarify comparative efficacy. Longer follow‐up would also help determine the duration of benefit and optimal maintenance intervals.

## 5 Conclusion

In this randomized split‐face trial, both PRP and PRF produced significant clinical improvements in periorbital hyperpigmentation, fine wrinkles, and mild hollowness, with comparable overall clinical efficacy, safety, and patient satisfaction. Biometric findings demonstrated a more stable reduction in melanin and erythema with PRF, suggesting that PRF may be more favorable when reduction of pigmentation is the primary treatment goal. In addition, PRF showed earlier improvement in selected elasticity parameters, indicating a potentially faster regenerative response. Both treatments resulted in measurable structural and clinical improvements; however, neither PRP nor PRF provided sufficient correction for severe hollowness, and multiple treatment sessions appear necessary to achieve and maintain optimal outcomes. The durability of clinical effects was similar between the two modalities, and periodic maintenance treatments every 3–4 months may be required to sustain regenerative benefits. Both treatments were well tolerated, although slightly greater discomfort was reported with PRP. Overall, these findings support the use of autologous platelet concentrates as effective, minimally invasive options for periorbital rejuvenation, while highlighting the need for larger, standardized studies with longer follow‐up to further define their comparative advantages and long‐term efficacy.

## Author Contributions

E.B., S.D., and M.A.N. contributed to the study by developing the research concept and design. E.B., R.Z., S.Z., and A.D. were responsible for trial execution, data collection, proposal drafting, securing ethical committee approval, and critically revising the manuscript. S.T.R. was conducting a literature review and critically drafting and revising the manuscript for significant intellectual content. R.Z. took part in the literature review, analysis, interpretation of revisions, prepared figures, and manuscript drafting. All authors have reviewed and approved the final version for publication and accept accountability for all aspects of the work. All authors contributed to the preparation and finalization of this article.

## Funding

This research was supported by the Skin and Stem Cell Research Center, Tehran University of Medical Sciences.

## Disclosure

The authors declare that no artificial intelligence–generated content was used in the conception, analysis, or interpretation of data in this manuscript.

## Ethics Statement

This study was approved by the Ethics Committee of the Iran University of Medical Sciences (approval number: IR.IUMS.FMD.REC.1402.382), under the project titled “Comparing the Safety and Effectiveness of Platelet‐Rich Fibrin (PRF) and Platelet‐Rich Plasma (PRP) in the Treatment of Periorbital Rejuvenation and Hyperpigmentation: A Randomized Double‐Blind Clinical Trial”, approved on 15 January 2024. Additional ethical approval was obtained from the Research Ethics Committee of Tehran University of Medical Sciences (approval number: IR.TUMS.MEDICINE.REC.1403.047) under the same project title, approved on 1 May 2024. The study was conducted in accordance with the principles of the Declaration of Helsinki.

## Consent

Written informed consent was obtained from all participants prior to enrollment. All collected data were kept confidential and analyzed anonymously. The authors have received permission to publish. Consent for publication is obtained from the patients.

## Conflicts of Interest

The authors declare no conflicts of interest.

## Data Availability

The data that support the findings of this study are available from the corresponding author upon reasonable request.
